# Vestibular evoked potentials (VsEPs) of cortical origin produced by impulsive acceleration applied at the nasion

**DOI:** 10.1007/s00221-014-4067-x

**Published:** 2014-08-20

**Authors:** Neil P. M. Todd, Aisha McLean, Aurore Paillard, Karolina Kluk, James G. Colebatch

**Affiliations:** 1 Faculty of Life Science, University of Manchester, Manchester, M13 9PL UK; 2 School of Psychological Sciences, University of Manchester, Manchester, M13 9PL UK; 3 Prince of Wales Clinical School and Neuroscience Research Australia, University of New South Wales, Randwick, Sydney, NSW 2052 Australia

**Keywords:** Impulsive acceleration (IA), Vestibular evoked myogenic potentials (VEMPs), Vestibular evoked potentials (VsEPs)

## Abstract

We report the results of a study to record vestibular evoked potentials (VsEPs) of cortical origin produced by impulsive acceleration (IA). In a sample of 12 healthy participants, evoked potentials recorded by 70 channel electroencephalography were obtained by IA stimulation at the nasion and compared with evoked potentials from the same stimulus applied to the forefingers. The nasion stimulation gave rise to a series of positive and negative deflections in the latency range of 26–72 ms, which were dependent on the polarity of the applied IA. In contrast, evoked potentials from the fingers were characterised by a single N50/P50 deflection at about 50 ms and were polarity invariant. Source analysis confirmed that the finger evoked potentials were somatosensory in origin, i.e. were somatosensory evoked potentials, and suggested that the nasion evoked potentials plausibly included vestibular midline and frontal sources, as well as contributions from the eyes, and thus were likely VsEPs. These results show considerable promise as a new method for assessment of the central vestibular system by means of VsEPs produced by IA applied to the head.

## Introduction

The vestibular apparatus plays a central role in balance and proprioception and mediates key reflexes for the stabilisation of posture and gaze. In mammals, the vestibular end-organs are naturally responsive to acceleration of the head, with the semicircular canals responsive to angular acceleration and the otoliths responsive to linear acceleration (e.g. Benson 1982). Animal experiments have shown that by applying linear or angular acceleration to the head, neurogenic vestibular evoked potentials (VsEPs) can be recorded from electrodes overlying the skull (Elidan et al. 1982; Jones et al. 1999, 2011; Plotnik et al. 1997). In humans, one of the ways that vestibular responses can be measured is by means of vestibular evoked myogenic potentials (VEMPs), which are manifestations of the vestibulo-collic and vestibulo-ocular reflex pathways (Colebatch et al. 1994; Rosengren et al. 2005; Todd et al. 2007). Various attempts have also been made to record VsEPs of neurogenic origin in humans, using a variety of short duration galvanic or acoustic stimulation (de Waele et al. 2001; Todd et al. 2003, 2008b, 2014a, b; McNerney et al. 2011). However, the use of intense linear or rotational acceleration to produce evoked electroencephalographic (EEG) responses in humans has historically been limited by stimulus artefact issues (Elidan et al. 1991).

In the last few years, the use of short, low-frequency impulsive accelerations (IA) applied to the skull has been developed, which is a stimulus likely to be selective for the utricle, especially when applied at the mastoid (Rosengren et al. 2009). When using this stimulus, well-defined responses can be measured in both ascending and descending projections to vestibulo-ocular (Todd et al. 2008a; Govender et al. 2011), vestibulo-collic (Rosengren et al. 2009) and vestibular-spinal systems (Laube et al. 2012). Such responses have been shown to be dependent on the direction of acceleration. This raises the possibility of applying such a stimulus to probe the central vestibular system by means of VsEPs. One of the practical difficulties in achieving this is that the usual stimulus location employed is the mastoid. However, when using an EEG cap, the mastoid is difficult to access without causing significant stimulus artefact. An alternative location is at the forehead, which is favoured by a number of workers using bone-conducted (BC) sound for evoking ocular VEMPs (OVEMPs) (e.g. Iwasaki et al. 2008). In the present study, therefore, we applied IA at the nasion for reasons of practicality.

A further complication that arises when applying such stimuli for the purpose of central activation is that vibration or acceleration impulses may also constitute significant tactile or proprioceptive stimulation, either through cutaneous receptors at the forehead or through stretch receptors in the musculature of the head and neck. It is necessary therefore to make use of a somatosensory control in order to establish whether any potentials evoked by IA at the forehead were due to the activation of vestibular receptors and were not somatosensory evoked potentials (SEPs). Although use of a mild galvanic stimulus applied at the nasion might seem suitable for this purpose, it would be prone to causing stimulus artefact and difficult using galvanic stimulation to reproduce the same timing pattern as that from an IA stimulus. We, therefore, chose to use the index fingers as alternative suitable locations for a control as their representation in the postcentral gyrus are close to those for the forehead. The aim therefore of the present study was to evaluate evoked potentials produced by IA of both positive and negative polarity at the nasion, compared with the same applied to the right and left index fingers.

## Methods

### Subjects

Twelve healthy participants were selected for this study (mean age = 27.5; SD = 7.21; three females and nine males) after screening for any neurological, vestibular or hearing impairments. The screening procedure included recording of both ocular and cervical VEMPs, to confirm normal vestibular function (Paillard et al. 2013), and audiograms to confirm normal hearing. Prior to any testing, all participants gave written informed consent according to the Declaration of Helsinki. The University of Manchester Ethics Committee approved the study. One participant was excluded from statistical analysis as data were not collected at all stimulus intensities. However, this participant’s data were included in the source analysis.

### Stimuli

**Fig. 1:**
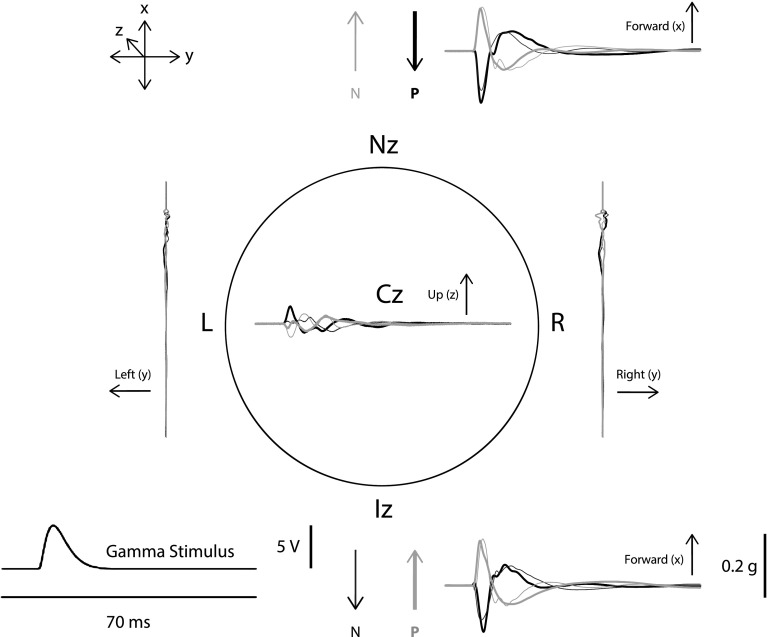
Acceleration responses of the head from stimulation at the nasion and inion with gamma pulses. The head is viewed from above and shows responses recorded at the nasion (Nz), inion (Iz), vertex (Cz), left (L) and right (R) mastoids using accelerometers placed normal to the recording site. The effects of four stimuli are shown averaged over four subjects, nasion positive (Nz P: outward displacement of the motor shaft causing acceleration of the head backwards, *bold black trace*), nasion negative (Nz N: inward displacement of the motor shaft, with initial acceleration of the head forwards, *thin grey trace*), inion positive (Iz P: outward displacement, with initial acceleration forwards, *bold grey trace*) and inion negative (Iz N: inward displacement, with initial acceleration backwards, *thin black trace*). Note that the forwards and backwards motions are very similar, whether produced by a negative/positive stimulus at the nasion/inion or vice versa. There is a relatively small motion in the* z* or* y* directions

IA stimuli were produced by applying a voltage shaped as third-order gamma distribution with 4-ms rise-time to a handheld vibrator (“Minishaker”, model 4810, Bruel & Kjaer P/L, Denmark) (Todd et al. 2008a). The minishaker was placed at the nasion, normal to the skull in the horizontal plane with a tonic pressure of about 10 N, via a cylindrical perspex rod of diameter of 2.5 cm and length of 9.2 cm. This arrangement produced a smoothly varying, approximately translational, whole-head acceleration pulse (see Fig. 1). All intensity values were referenced to a peak amplitude of 0.2 g. As there was a tonic pressure, a reversal of voltage polarity gave rise to a head acceleration response which was opposite in direction and approximately equal in peak amplitude. Thus, a positive polarity voltage produced a backwards motion and a negative polarity a forwards motion. As frequency content of this stimulus is low, i.e. less than 100 Hz, it did not produce a significant sound component. The minishaker was held so that it did not block a participant’s view of the movie.

### VsEPs

VsEPs were recorded with subjects comfortably seated with their gaze directed straight ahead to a screen displaying silent movies at a viewing distance (about 70 cm). Stimuli were presented at intervals varying randomly between 600 and 1,000 ms, up to a total of 400 stimuli per trial. Evoked potentials (EPs) were recorded for a range of intensities, i.e. −6, −9, −12, −15, and −18 dB re 0.2 g for both forward and backward motions. The recordings were made in two sessions. In the first session, stimulation was applied on the nasion. In the second session, a somatosensory control condition was recorded. This consisted of sequentially stimulating the left and right index fingers with the minishaker at a fixed intensity (−6 dB re 0.2 g). The participant’s hand was placed in a relaxed, supported position on a table at their side with the index finger resting on the minishaker placed on the table next to the hand. A positive or negative polarity voltage produced a corresponding upwards or downwards motion of the finger.

EEG was recorded using a 64-channel EEG system (Biosemi, Inc., USA). Additional electrodes were also placed below each eye (i.e. infra-ocular electrodes, IO1 and IO2), at deep frontal locations (F9 and F10) and at the ear-lobes (A1 and A2). Electrode offset [i.e. running average of the voltage measured between the reference electrode (CMS) and each active electrode] was maintained below 20 µV. EPs were obtained over an epoch of 350 ms, from 50 ms before to 300 ms following the stimulus onset, with a band-pass of between 0.16 Hz and 1 kHz. Amplitudes and latencies were measured at responses peaks, after referencing to linked earlobes and further band-pass filtering between 1 and 200 Hz using Neuroscan software (v4.3, Neuroscan, USA). As the actual motion produced by the stimulus was small, there was no significant motion stimulus artefact.

### Source modelling

BESA software (version 5.1 MEGIS Software GmbH, Germany) was used for dipole modelling. The standard four-shell elliptical head approximation was employed with the following parameters. The radial thickness of the head, scalp, bone and CSF was 85, 6, 7 and 1 mm, respectively, with conductivities set to 0.33, 0.33, 0.0042 and 1.0, respectively. Prior to conducting the source analysis, changes in the global field power with intensity were also evaluated in order to determine the appropriate fitting epoch. It was found appropriate to model the same interval as was used for SEPs, i.e. 20 to 80 ms. We adopted a modelling strategy from previous occasions of starting from simple dipole models fitted by a genetic algorithm and then gradually building the complexity. In particular, if a source located to an ocular region, it was replaced by a symmetrical pair of regional sources. Regional sources are appropriate to model the complexity of the (known) activation of the bilateral extra-ocular eye muscles (EOM) in conjunction with the retinal corneal dipole (RCD) associated with eye movement.

## Results

### Evoked potentials from index finger stimulation

**Fig. 2:**
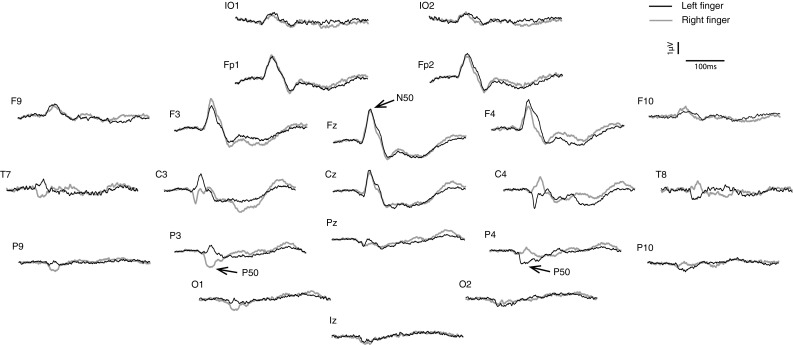
Average grand means of SEPs produced by IA at the *right* and *left* index fingers. Waveforms were averaged across positive and negative polarities as there was no discernible difference between the two polarities

**Fig. 3:**
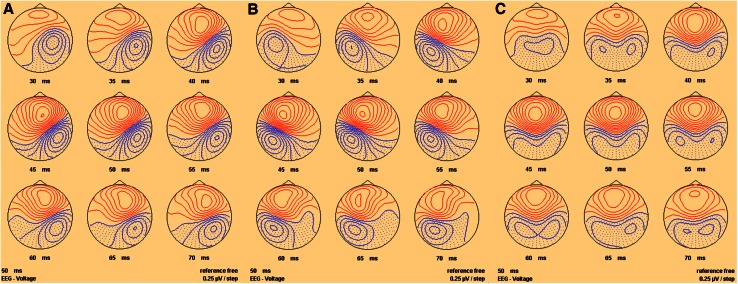
Sequential top meridian projection of scalp map of SEPs produced by IA at **a** the left finger, **b** the right finger and **c** left and right fingers combined. *Red* indicates a negativity, *blue* a positivity

Applying IA to the left and right index fingers yielded a response which did not vary with the polarity of the applied acceleration, i.e. the mean evoked response for +ve or −ve polarity was not noticeably different on visual inspection. For this reason, responses from positive and negative polarity stimulation were averaged together for each finger, and the results of this are illustrated in Figs. 2 and 3. Consistent with the prior literature on SEPs, the response here was characterised by a single large peak in potential at about 50 ms which on the scalp map exhibited a single-phase reversal around the central electrodes contralateral to the side of stimulation. Thus, in postcentral leads, a P50 was maximal at CP3 and CP4 for left- versus right-side stimulation, respectively. For precentral leads, a corresponding N50 maximum was located close to Fz for left and right finger stimulation.

### Evoked potentials from nasion stimulation

**Fig. 4:**
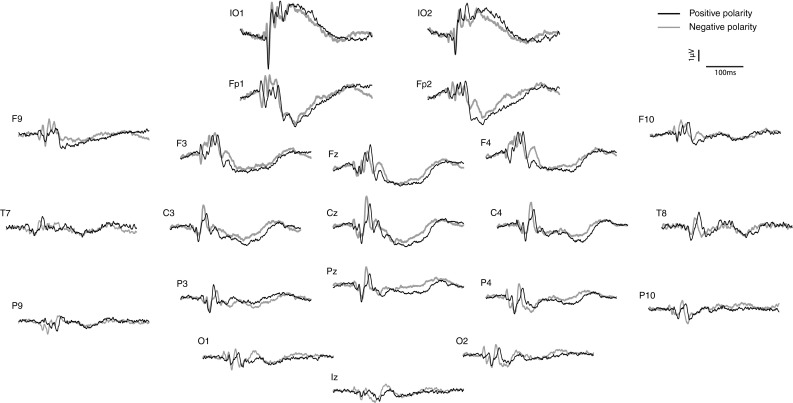
Grand means of evoked potentials produced by IA applied to the nasion, comparing positive and negative polarity stimulation

**Fig. 5:**
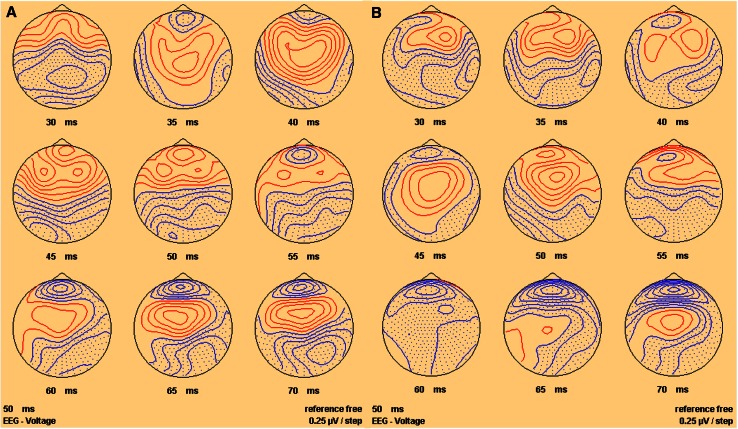
Sequential top meridian projection of scalp map of VsEPs produced by IA at nasion with **a** positive polarity IA and **b** negative polarity IA. *Red* indicates a negativity, *blue* a positivity

In contrast to stimulation at the fingers, the response to IA applied to the nasion exhibited multiple and dynamic foci in the scalp map and a distinct series of peaks whose polarity and latency were dependent on the polarity of the stimulus acceleration. It should be emphasised that these polarity and latency shifts in the response are not due to any differences in latency of the stimulus with the changing polarity, which were negligible, as is clear from the accelerometry measured at Iz and Nz (Fig. 1), but are an intrinsic property of the response itself. The peaks were labelled according to latency at which they appeared at FCz in response to the highest stimulus intensity (−6 dB re 0.2 g). Thus, in response to positive polarity IA, five positive and negative deflections were recorded, i.e. an N26, P30, N40, P55 and N65, and for negative polarity IA, six peaks, i.e. P26, N35, P40, N50, P60 and N78. The scalp distributions of these waveforms are shown in Figs. 4 and 5. In addition to the complex response shown in central leads, the nasion evoked response also exhibited large amplitude components in infra-ocular and prefrontal leads, which were also polarity dependent.

### Comparison of finger versus nasion evoked responses

**Fig. 6:**
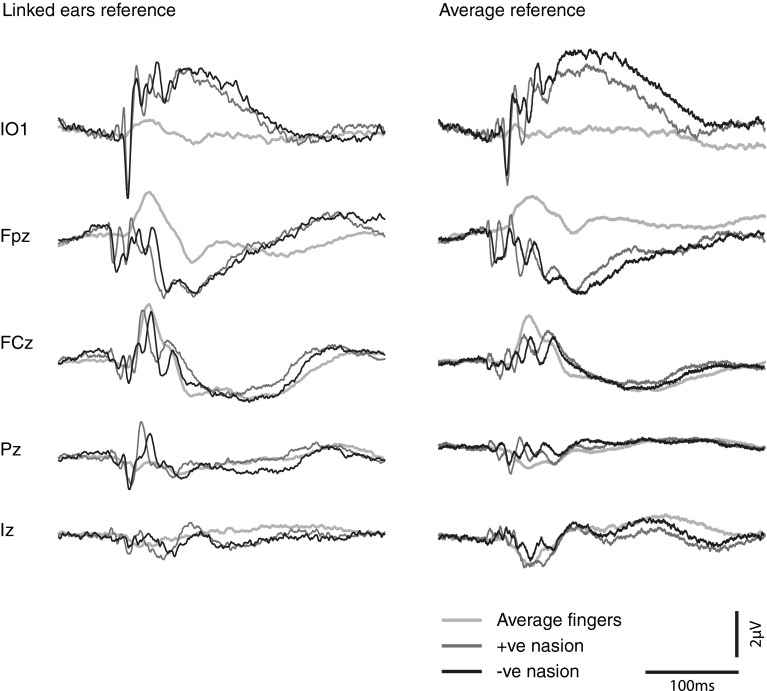
Recordings from IO1 and central electrodes Fpz, FCz, PZ and Iz showing the grand means of evoked potentials produced by IA of positive and negative polarity at the nasion, compared to evoked potentials elicited by the average of positive and negative IA applied to right and left index fingers (to simulate simultaneous stimulation). Waveforms with linked ears (*left*) and average reference (*right*) are both shown for comparison

In order to compare directly finger and nasion evoked responses, waveforms from selected leads, i.e. at IO1, Fpz, FCz, Pz and Iz, are illustrated in Fig. 6. An average of the potentials evoked by left and right finger stimulation was used in this comparison. As can be seen from this figure, the three waveforms generated by positive polarity IA at the nasion, negative polarity IA at the nasion and IA applied to the finger are distinct at each of the electrodes. These differences are apparent regardless of whether a linked ears reference or an average reference is used. The single peak evoked by average finger stimulation is largest at Fpz and FCz, and the polarity becomes reversed between FCz and Pz. For IA applied to the nasion, the response waveforms show some evidence of a polarity reversal according to the polarity of the stimulus. This effect is most obvious at FCz and IO1 and is less apparent when using a linked ears reference.

### Effects of polarity and intensity on waveform and global field power

**Fig. 7:**
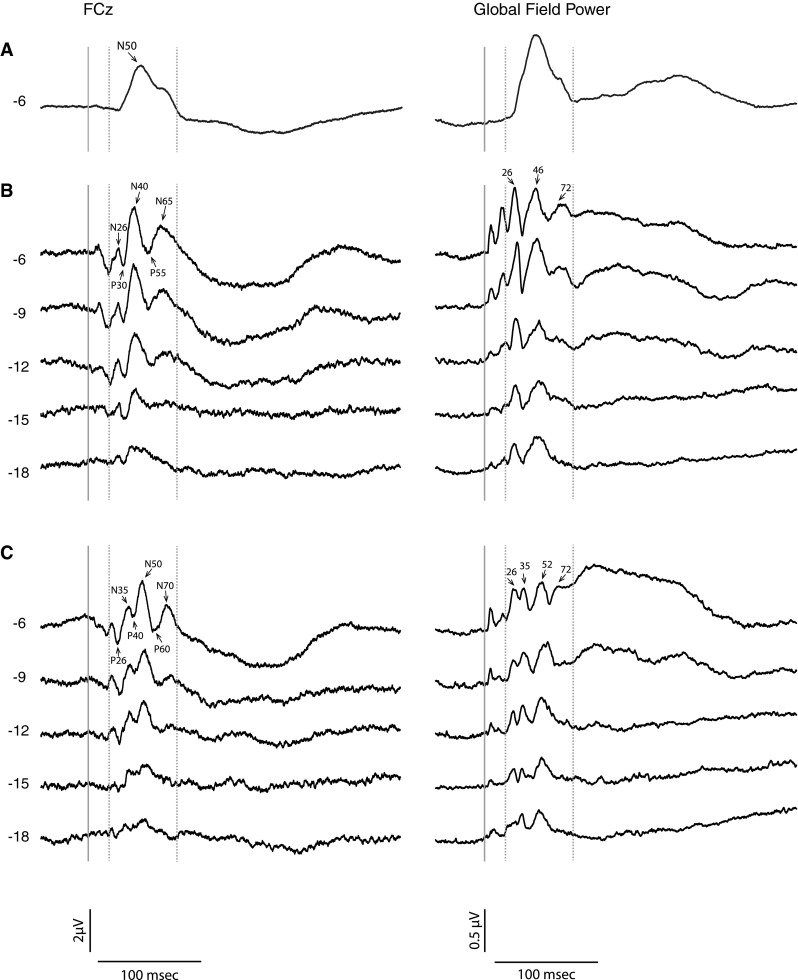
Waveforms recorded at FCz compared to the global field power for each of the stimulus intensities from **a** average of finger stimulation at the highest stimulus intensity, **b** positive and **c** negative polarity IA

Prior to conducting a source analysis, waveforms and global field power (GFP) for each condition were examined as a function of intensity (see Fig. 7). As above for the individual potentials at FCz, the GFP of the finger evoked response is characterised by a single lobe with a peak at about 50 ms. In contrast, for nasion evoked responses, a series of smaller lobes can be identified which are polarity dependent. At the highest intensity for the positive polarity, the sequence of five peaks corresponds to three lobes in the GFP at 26, 46 and 72 ms, while for the negative polarity the six peaks correspond to four lobes at 26, 35, 52 and 72 ms. In addition to the effect of polarity at the nasion, the waveform morphology and GFP structure change with stimulus intensity. In particular, the later components appear to have a higher threshold. Thus, for the positive polarity, the P55 and N65 peaks are not identifiable at the lowest intensity, i.e. −18 dB, while for the negative polarity, the P60 and N78 peaks become unidentifiable by eye at a stimulation intensity of −15 dB. Within the GFP, the last component at 72 ms similarly drops out at the lower intensities. This difference in threshold property was used to label the potentials “early” or “late”.

### Statistical analysis

ANOVA was performed to compare statistically the effect of intensity on the amplitude and latency of the stimulus applied to the nasion using within-subject factors of intensity and wave. As there was a clear difference in threshold between the early and late waves, the analysis was carried out independently for these cases for both conditions (negative and positive polarity IA). Thus, for the positive polarity, the N26, P30 and N40 latencies and corresponding peak–peak N26–P30 and P30–N40 amplitudes were considered as early, while the P55 and P65 latencies and N40–P55 and P55–P65 amplitudes were considered as late. For the negative polarity stimulus, the P26, N35, P40 and N50 latencies and corresponding peak–peak P26–N35, N30–P40 and P40–N50 amplitudes were considered as early, while the P60 and N70 latencies and N50–P60 and P60–N70 amplitudes were considered as late.

**Fig. 8:**
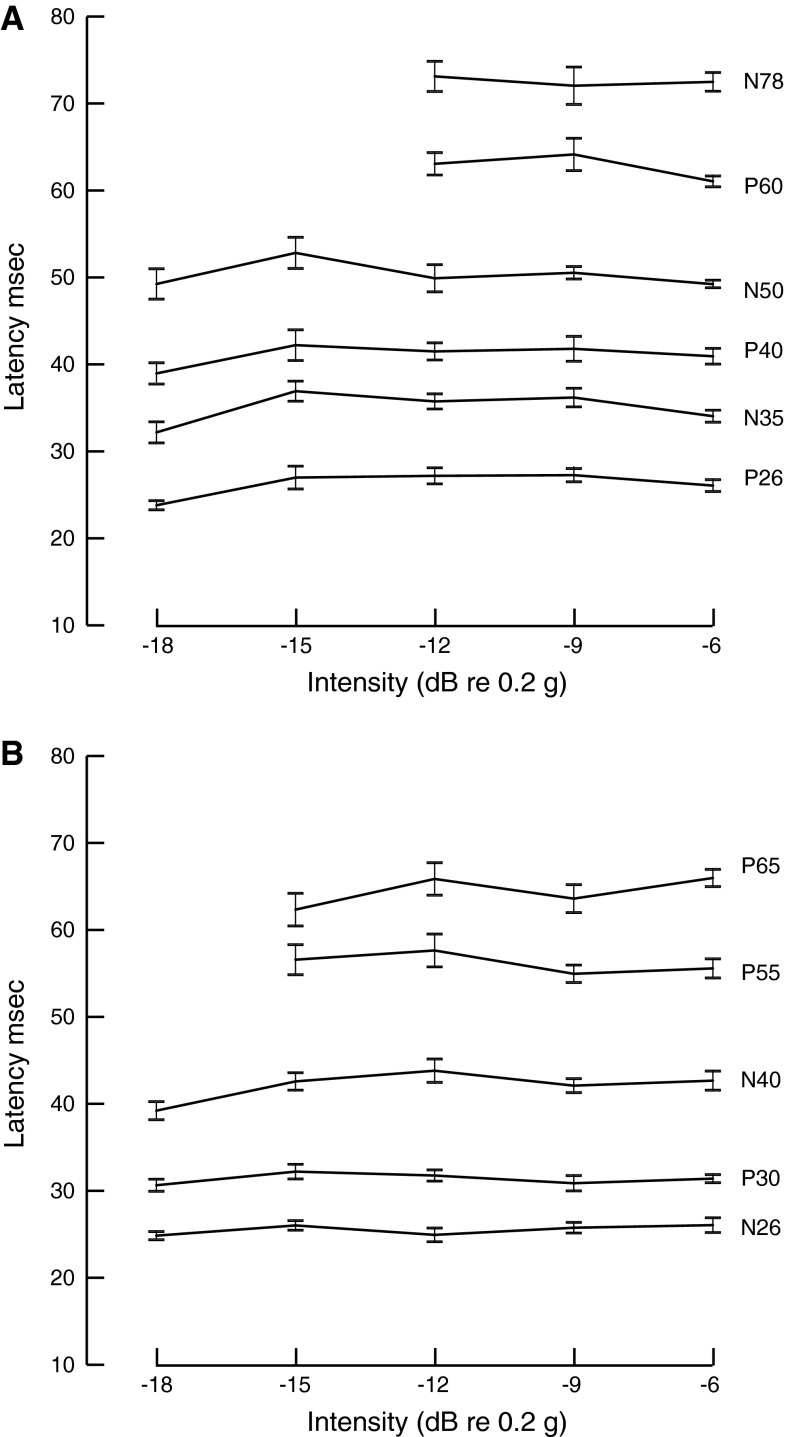
Intensity dependence of peak latencies from **a** negative polarity IA and **b** positive polarity IA. All values are marginal means where *error bars* show standard error

For the latency analysis (Fig. 8), for both positive and negative polarity cases, there was a main effect of intensity for the early but not the later peaks (Table 1). Thus, the early components tended to increase in latency from the lowest intensity and then become shorter as intensity increased. In contrast, the later components did not change latency with intensity. As would be expected, there were main effects of wave, but no interactions, so that the early and late components all showed similar behaviours.

**Table 1:** Within-subject effects from a repeated measures ANOVA with peak latency as dependent variable and within-subject factors of intensity and wave

IA polarity	Latency	Effect	Degrees of freedom	F-ratio	*p* value
Positive	Early	Intensity	4.40	3.1	**<0.05**
		Wave	2.20	334.6	**<0.001**
		Intensity × wave	8.80	2.2	0.11
	Late	Intensity	3.30	0.8	0.47
		Wave	1.10	77.9	**<0.001**
		Intensity × wave	3.30	3.3	0.06
Negative	Early	Intensity	4.40	3.6	**<0.05**
		Wave	3.30	542.2	**<0.001**
		Intensity × wave	12.120	0.6	0.64
	Late	Intensity	2.20	0.4	0.68
		Wave	1.10	160.9	**<0.001**
		Intensity × wave	2.20	1.6	0.22

For the +ve polarity stimulus, the early waves were N26, P30 and N40 and the late waves P55 and N65. For the −ve polarity stimulus, the early waves were P26, N35, P40 and N50 and the late waves P60 and N78. Greenhouse–Geisser *p* values are given and values <0.05 are highlighted in bold

**Fig. 9:**
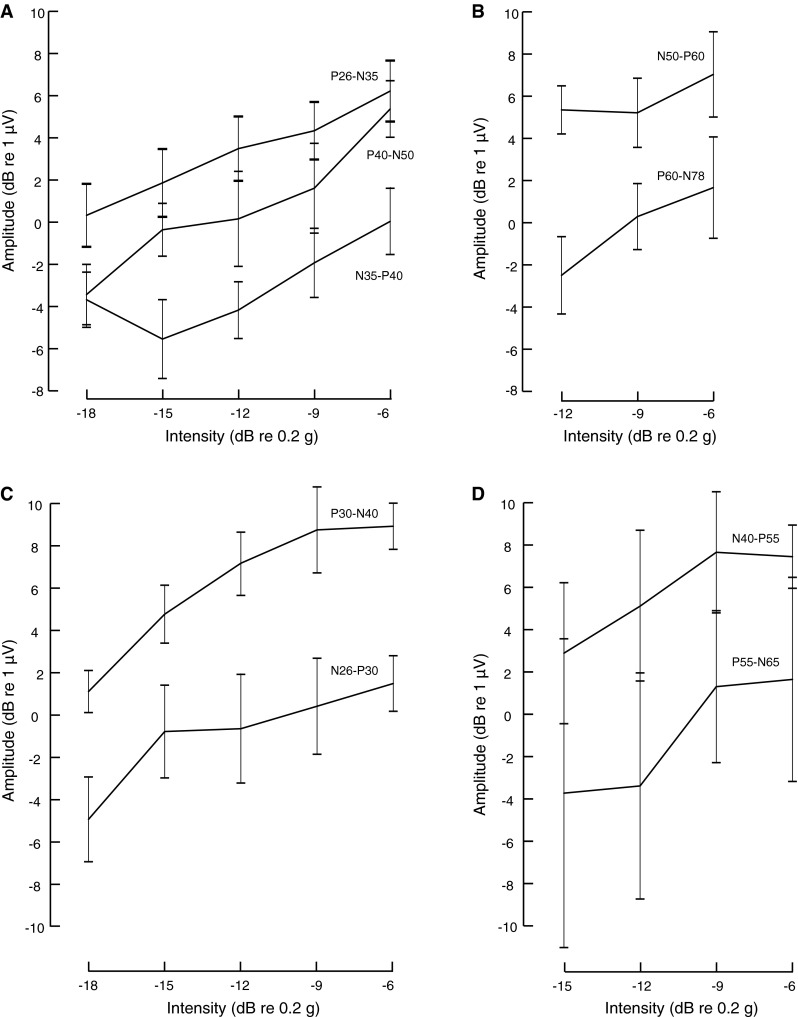
Intensity dependence of **a** early peak–peak amplitudes from negative polarity IA, **b** late peak–peak amplitudes from negative polarity IA, **c** early peak–peak amplitudes from positive polarity IA and **d** late peak–peak amplitudes from positive polarity IA. All values are marginal means where *error bars* show standard error

When applied to log-transformed peak-to-peak amplitude values, with the same factors of intensity and wave (Fig. 9; Table 2), for positive polarity stimulation, there was a significant main effect of intensity on both early and the later peak amplitudes. For negative polarity stimulation, there was a significant effect of intensity on the early but not on the later peak amplitudes.

**Table 2:** Within-subject effects from a repeated measures ANOVA with peak–peak amplitude as dependent variable and within-subject factors of intensity and wave

IA polarity	Latency	Effect	Degrees of freedom	F-ratio	*p* value
Positive	Early	Intensity	4.40	8.1	**<0.005**
		Wave	1.10	22.0	**<0.005**
		Intensity × wave	4.40	0.9	0.47
	Late	Intensity	3.30	5.3	**<0.05**
		Wave	1.10	9.4	**<0.05**
		Intensity × wave	3.30	0.5	0.58
Negative	Early	Intensity	4.40	6.2	**<0.005**
		Wave	2.20	14.3	**<0.005**
		Intensity × wave	8.80	0.8	0.54
	Late	Intensity	2.20	1.9	0.19
		Wave	1.10	16.4	**<0.005**
		Intensity × wave	2.20	0.8	0.46

For the +ve polarity stimulus, the early peak–peak waves were N26–P30 and P30–N40 and the late peak–peak waves N40–P55 and P55–N65. For the −ve polarity stimulus, the early peak–peak waves were P26–N35, N35-P40 and P40–N50 and the late peak–peak waves N50–P60 and P60–N78. Greenhouse–Geisser *p* values are given and values <0.05 are highlighted in bold

As both positive and negative polarity early evoked responses showed a significant linear contrast (*F*_(1,10)_ = 37.4, *p* < 0.001 and* F*_(1,10)_ = 26.1, *p* < 0.001, respectively), but no higher order contrasts, the amplitude-intensity functions obeyed approximately a simple power law with respect to the stimulus. The early components also yielded significant main effects of wave so that for positive stimulation the P30–N40 component was largest and for negative polarity stimulation the P26–N35 was largest.

### Source analysis

**Fig. 10:**
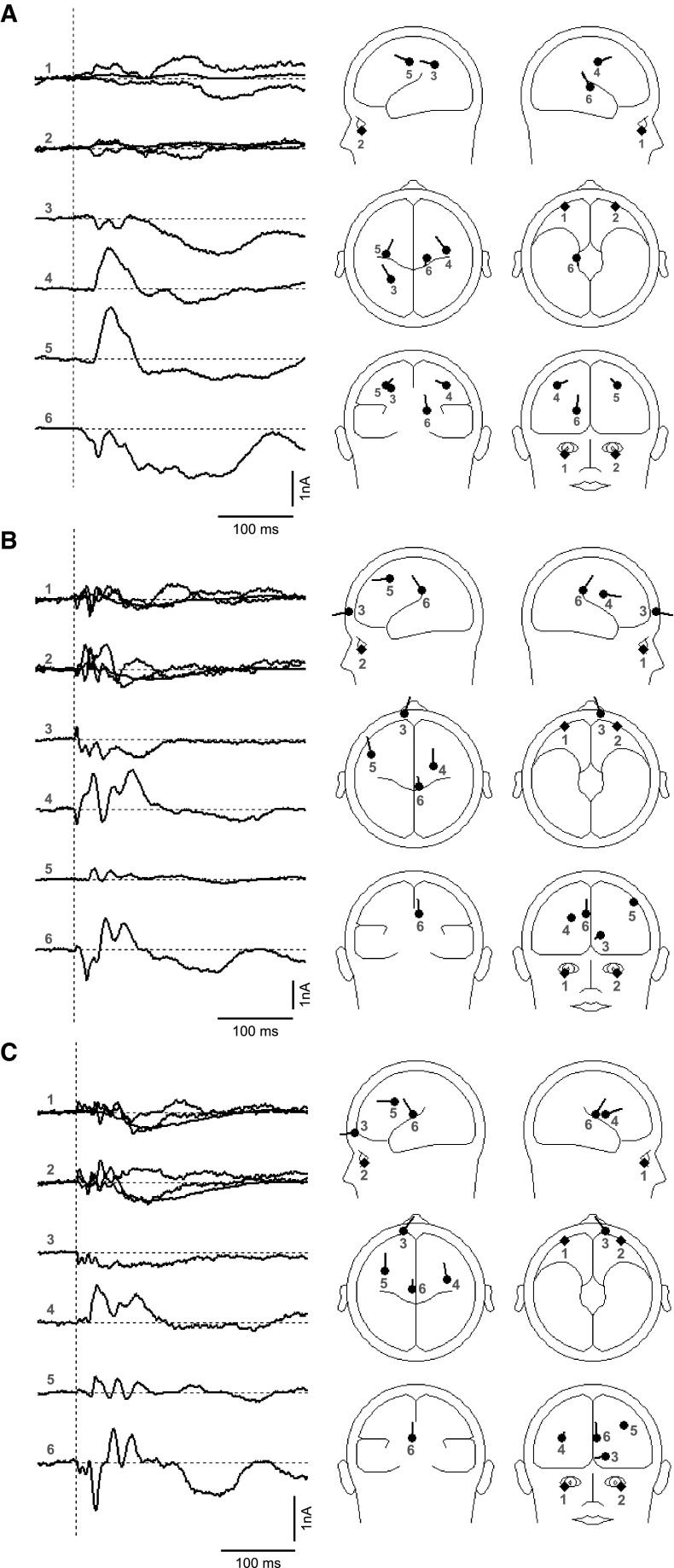
Source analyses of **a** combined left and right finger evoked SEP, **b** positive polarity nasion evoked VsEPs and **c** negative polarity nasion evoked VsEPs. (*Left*) Source current waveform (*right*) source locations mapped onto the head model

Although extensive analyses were conducted starting from the simplest models, we report here only the final outcomes which employed a symmetrical pair of regional sources (RSs) for the ocular components along with three or four dipoles. These outcomes are illustrated in Fig. 10, and the Talairach coordinates are given in Table 3.

**Table 3:** TTCs for BESA applied to finger and nasion evoked potentials

Model	Condition	X	Y	Z	RV	Region	Origin
3DP + pairRS	Fingers	−34	−15	46	0.8	L parietal/frontal	BA3/4/2
		41	−12	46		R parietal/frontal	BA4/6/3
		14	−17	13		R thalamic	VL/LP/MD/LD/VPL/VPM
		±30	62	−40		Ocular	L + R EOG
4DP + pairRS		−34	−17	46	0.7	L parietal/frontal	BA4/3
		41	−11	46		R parietal/frontal	BA4/6/3
		17	−19	11		R thalamic	LP/Pul/VL/VPL/VPM/LD/MD
		−27	−52	38		L parietal	SPL/IPL/BA 7
		±31	61	−39		Ocular	L + R EOG
3DP + pairRS	+ve Nz	5	−32	33	9	Midline	CG/BA 31/23
		24	−3	31		R frontal	R BA 6
		−13	74	16		Ocular/scalp	Frontalis?
		±32	62	−37		Ocular	L + R OVEMP/EOG
4DP + pairRS		2	−34	29	8	Midline	CG/BA 31/23
		35	−6	31		R frontal	R BA 6
		−52	12	54		L frontal	MFG/BA 6
		−6	73	13		L prefrontal	SFG/BA 10
		±32	62	−36		Ocular	L + R OVEMP/EOG
3DP + pairRS	−ve Nz	−3	−19	19	8	Midline	Thalamus M/A
		30	1	27		R frontal	R BA 6
		−16	75	7		Ocular/scalp	Frontalis?
		±33	61	−37		Ocular	L + R OVEMP/EOG
4DP + pairRS		−5	−16	20	7	Midline	Thalamus M/A/LD
		38	−1	22		R frontal	Ins/PreCG/IFG/BA 13/6/9
		−39	9	40		L frontal	MFG/PreCG/BA 9/6/8
		−16	70	6		L prefrontal	SFG/MFG/BA 10
		±34	61	−37		Ocular	L + R OVEMP/EOG
4DP + pairRS		−6	−9	19	7	Midline	Thalamus A/caudate
		42	12	30		R frontal	MFG/PreCG/IFG/BA 9/46
		−39	34	40		L frontal	MFG/BA 8/9
		−37	−42	−8		L temporal	FusG/ParahippG/BA 37/19
		±29	62	−40		Ocular	L + R OVEMP/EOG

*BA* Brodmann area, *CG* cingulate gyrus, *DP* dipole, *EOG* electro-oculogram, *FusG* fusiform gyrus, *Ins* insula, *IFG* inferior frontal gyrus, *L* left, *MFG* medial frontal gyrus, *ParahippG* parahippocampal gyrus, *PreCG* precentral gyrus, *RS* regional source, *R* right, *RV* residual variance, *SFG* superior frontal gyrus, *TTC* talairach–tournoux coordinates

Considering first the combined finger response, bilateral ocular components are present, which corresponding to the eye movements associated with watching a movie during the recording. No additional filtering from the original (0.16–1,000 Hz) was employed, so any eye movement EOG was not removed. Other than the EOG components, for the three-dipole case, the sources located to bilateral parieto–frontal areas, very close to the central sulcus (BA 3 and 4), and an additional source in the right thalamus. With the addition of a fourth dipole, a source was also located in the left parietal lobe. The residual variance (RV) for these models was between 0.7 and 0.8 %.

For the nasion response, positive and negative polarity cases are modelled similarly, with a RV of between 7 and 8 %. Both include an ocular pair, a deep midline source, bilateral frontal sources and a midline source close to the nasion. For positive polarity stimulation, the deep midline source was located within the cingulate cortex (BA 31 and 23) but within the thalamus for negative polarity stimulation. The bilateral frontal sources for positive stimulation corresponded to BA 6, within the precentral gyrus or medial frontal gyrus (MFG). For negative polarity stimulation, these extend to BA 9 and 8, including inferior frontal gyrus (IFG), and to anterior insula (BA 13). The anterior midline source appeared to be outside the brain for the three-dipole model cases, but located to left superior or middle frontal gyri (BA10) when four sources were employed. With negative polarity stimulation, a left temporal source was also indicated.

## Discussion

The morphology of the waveform evoked by application at the fingers is invariant with stimulus polarity and shows the expected contralateral focus for a somatosensory projection. Consistent with previous literature on SEPs in response to tactile pulses (acceleration) applied to the index fingers, the main peak recorded in this study following finger acceleration resembles the contralateral N50/P50 component described by Hämäläinen et al. (1990), which was believed to be generated in the primary somatosensory cortex. Source analysis using a four-dipole model indicated a large contribution from a radial source in the postcentral gyrus contralateral to the stimulated hand (Srisa-an et al. 1996). This peak has also been attributed to both radial and tangential components originating from two perirolandic dipoles in a five-dipole model (Barba et al. 2002). The analysis presented here of the combined finger evoked potentials located sources within a pericentral area bilaterally. These locations appear to be somewhat anterior of the primary somatosensory cortex, which may suggest limitations in the resolution of the source analysis method employed. However, Barba et al. (2002) also reported frontal sources. Our analysis is thus consistent with the prior literature suggesting both somatosensory and motor generators of the N50/P50.

On the somatotopic map, the area represented by the index finger is adjacent to the area represented by the forehead. Therefore, if the evoked potentials elicited by the application of IA to the nasion were primarily somatosensory, with no vestibular component, the waveform would resemble that elicited by acceleration applied to the index fingers combined. However, the grand means of EEG activity in response to IA at the nasion show quite distinct waveforms and are polarity dependent. This implies that IA applied to the nasion is activating a different set of receptors than when applied to the finger. As noted in the methods section, the stimulus employed did not produce a significant sound and the actual motion of the shaker was small. It is unlikely therefore that the response includes either auditory or visual components. As described above, IA has been demonstrated by means of VEMPs to be an effective vestibular stimulus when applied to the skull (Todd et al. 2008a; Rosengren et al. 2009). It is likely therefore that the nasion evoked responses recorded here are vestibular in origin and should be considered as VsEPs. Although the IA would also have activated muscle receptors in the neck (Halmagyi et al. 1995), the contribution of muscle afferents to evoked potentials is small (Allison et al. 1991).

As would be expected from the prior literature, a significant contribution to VsEPs is ocular in origin, and this can be observed in the large responses obtained in the infra-ocular electrodes. These appear to be composed of short-latency, likely OVEMP components, and longer latency contributions which are likely EOG caused by evoked eye movement. The evoked EOG component is manifested in the phase reversal between the IO leads and Fpz. The source analysis confirmed the ocular contribution represented by the pair of regional sources in which the early OVEMP and later EOG contributions can be discriminated.

The non-ocular cortical sources are quite distinct from those obtained for the SEP analysis. All solutions involved a deep midline source which may be representing cingulate cortex, a medial thalamus area or basal ganglia. Some caution is required in the interpretation of this source given the limitations in accuracy of the method, especially for deep sources, and the histology of the thalamus may limit its contribution to scalp-recorded potentials. However, activity within the cingulate gyrus is consistent with the literature on vestibular cortical projections in which a “vestibular cingulate region” has been identified in primate studies (Guldin and Grüsser 1998). In humans, the vestibular cingulate area extends from anterior cingulate to middle and more posterior cingulate areas (Lopez and Blanke 2011). In our case, the source was located in anterior portions of BA 23 and 31 (posterior cingulate). A number of imaging studies using caloric and galvanic vestibular stimulation have observed activity in this area (Lobel et al. 1998; Suzuki et al. 2001; Fasold et al. 2002). Todd et al. (2014a, b) also reported a cingulate source using air-conducted sound stimulation.

In addition to the deep midline source, bilateral frontal cortex was implicated in the source analysis, corresponding approximately to the vestibular premotor areas encompassing BA 6, 8 and 9 (Lopez and Blanke 2011). As above, these areas are commonly activated in imaging studies using caloric and galvanic vestibular stimulation (Bense et al. 2001; Fasold et al. 2002). Todd et al. (2008b) also found evidence of bilateral frontal activity in a source analysis of VsEPs from air- and bone-conducted sound.

The analysis also implicated an anterior midline source, which may represent activity in prefrontal cortex corresponding to BA 10 within superior or medial frontal gyri. This area is less commonly observed in imaging studies (Stephan et al. 2005), but is close to source 2 of de Waele et al. (2001). The source may also represent an extra-cortical source possibly from the frontalis muscle in response to cutaneous stimulation at the nasion. One solution to negative polarity stimulation also implicated the parahippocampal gyrus BA 37, which is also established as a vestibular receptive region (Vitte et al. 1996; Suzuki et al. 2001; Lopez and Blanke 2011).

Thus, in sum, the source analysis conducted of the nasion evoked VsEPs is both distinct from the sources responsible for finger evoked SEPs and plausibly vestibular when compared with those found in the imaging studies. Given the strong indication of parieto-insular vestibular cortex in the prior imaging studies, the predominantly frontal activation found here may again suggest limitations of the method. However, both galvanic and caloric stimulation give rise to strong activation of the canals. In contrast, our stimulus is more likely to be specific for the utricle and the sources found here may be indicative of this. Further studies will be required to clarify this issue.

The VsEPs produced by IA at the nasion decrease in amplitude as stimulus intensity decreases and obey approximately a power law. We have shown that the VEMP threshold to IA was between about −8 to −26 dB re 0.2 g, depending on the VEMP peak considered (Paillard et al. 2013). The very earliest peaks of the VEMP waveform had significantly higher thresholds than the later peaks, which may be related to the differences that we observed here for the early and late VsEP waves. The lowest threshold was about 8 dB below the lowest stimulation intensity used here to record the early components, at which a response was clearly still observable in the GFP (Fig. 4). Further studies will be required to compare VsEP thresholds as assessed by means GFP with those from the VEMP.

In addition to the similar threshold and intensity dependence of amplitude properties, VsEPs evoked by IA also share a property with VEMPs in being polarity dependent, with either five or six peaks recordable within the analysis window of 20–80 ms that we considered. It has previously been argued that direction dependence of IA evoked VEMPs supports an otolithic origin, as the morphological polarised hair cells within otolith organs are sensitive to the direction of stimulation, and this argument could be applied here to suggest that the IA evoked VsEPs are also otolithic. However, some caution is required in this interpretation. The OVEMPs induced by IA may last up to 30 ms, and a contribution from these myogenic waves cannot be excluded. In the prior VEMP studies using IA, the preferred place of stimulation was the mastoid, to prevent angular acceleration of the head about the z- or x-axis (Rosengren et al. 2009). Due to constraints of using the high-density EEG cap, it was not possible to use the mastoid in the present study; thus, a contribution from the semicircular canals cannot be ruled out here. For future clinical studies, the use of a smaller, select number of channels, mastoid stimulation will be preferable. It is likely that the earlier waves with the lowest thresholds should prove most reliable for such clinical work. These are the N26, P30 and N40 for Nz positive stimulation, and P26, N35, P40 and N50 for Nz negative stimulation. Further experiments will be also required, especially making use of a vestibular patients and with alternative control conditions, to confirm the vestibular dependency of the potentials suggested by the present data.
